# 超高效液相色谱-傅里叶变换离子回旋共振质谱法解析溶解性有机质的化学多样性

**DOI:** 10.3724/SP.J.1123.2023.03012

**Published:** 2023-08-08

**Authors:** Chao MA, Hongxing NI, Yulin QI

**Affiliations:** 1.天津大学表层地球系统科学研究院,地球系统科学学院,天津 300072; 1. Institute of Surface-Earth System Science, School of Earth System Science, Tianjin University, Tianjin 300072, China; 2.中化资产管理有限公司,北京 100045; 2. Sinochem Asset Management Co., Ltd., Beijing 100045, China; 3.物质绿色创造与制造海河实验室,天津 300192; 3. Haihe Laboratory of Sustainable Chemical Transformations, Tianjin 300192, China

**Keywords:** 超高效液相色谱, 傅里叶变换离子回旋共振质谱, 溶解性有机质, 可视化表征, ultra performance liquid chromatography (UPLC), Fourier transform ion cyclotron resonance mass spectrometry (FT-ICR MS), dissolved organic matter, visualization

## Abstract

采用傅里叶变换离子回旋共振质谱(FT-ICR MS)结合四极检测技术,对水体、气溶胶和土壤样品中溶解性有机质(DOM)进行在线超高效液相色谱(UPLC)-质谱分析。3种环境样品主要组成为强极性含盐化合物、富氧低饱和度的单宁酸类化合物、低氧高饱和度的木质素类化合物及蛋白/氨基糖类化合物。UPLC-FT-ICR MS适合分析DOM的分子组成,在水体、气溶胶和土壤DOM中能够有效匹配分子式的质谱峰数目分别为12027、15593和8029个。质谱数据经可视化处理后发现,水体样品的特有组分主要出现在0.1<O/C<0.5、1.0<H/C<1.7区域,为低氧高饱和度的木质素组分;气溶胶样品的特有组分主要出现在0.4<O/C<1.0、1.5<H/C<2.0区域,属于碳水化合物的分布范围;土壤样品在不同极性洗脱段有不同的特有组分,亲水段的特有组分主要出现在0.6<O/C<1.0、0.5<H/C<1.0区域,为单宁酸类化合物,而疏水段的特有组分主要为木质素类化合物。本研究以探索的方式,提出了采用UPLC-FT-ICR MS分析环境体系DOM的方法,选用不同极性的流动相利用UPLC对环境样品中的DOM进行分离,并结合高分辨质谱分析,表征了DOM分离为不同极性组分后的分子组成以及其可能的化合物类型,为揭示更全面的DOM分子组成提供了技术支持。

## Abstract (en)

Dissolved organic matter (DOM) is a highly complex and heterogeneous mixture that exists in various environments, including rivers, oceans, soils, and atmospheric aerosols. DOM plays a crucial role in biogeochemical cycles and significantly influences the environment by regulating water quality, changing the climate, and transporting pollutants. Therefore, clarifying the detailed molecular composition of DOM is essential to obtain a better understanding of its physical and chemical properties, thereby enabling further elucidation of its biogeochemical behavior.

In this study, Fourier transform ion cyclotron resonance mass spectrometry (FT-ICR MS) combined with quadrupole detection (QPD) was used to conduct the online ultra performance liquid chromatography (UPLC)-MS analysis of DOM in water, aerosol, and soil samples collected in Tianjin, China. The samples were extracted with pure water and filtered through a glass fiber membrane (0.45 μm). The DOM in the samples was then enriched by solid-phase extraction (SPE) and redissolved in water-acetonitrile (1∶1, v/v) at mass concentration of 200 mg/L for the LC-MS experiments. The mobile phases used for UPLC were water containing 0.1% (v/v) formic acid (A) and acetonitrile containing 0.1% (v/v) formic acid (B). The gradient elution procedure was as follows: 0-5 min, 0B; 5-11 min, 0B-95%B; 11-25 min, 95%B; 25-28 min, 95%B-0B; 28-30 min, 0B. The flow rate was 0.1 mL/min, and the injection volume was 10 μL. The UV wavelength was set at 274 nm. MS detection was performed in negative electrospray ionization (ESI(-)) mode with a capillary voltage of 5.0 kV, and the MS data were collected in broadband (*m/z* 150-1000) and QPD modes. The transient data size was set to 2M, the free induction decay signal length was 0.74 s, and the ion accumulation time was 0.030 s.

Four chromatographic peaks were observed in the chromatograms. The first peak was identified as salt adduct compounds containing sodium formate. The three other peaks contained complex components, such as oxygen-rich, unsaturated tannin-like compounds, as well as low-oxygen, highly saturated lignin-like and protein/amino-like compounds. UPLC-FT-ICR MS was suitable for assigning the detailed elemental compositions of the DOM samples. UPLC effectively improved the ionization efficiency of difficult-to-ionize compounds and enhanced the detection accuracy of MS. Indeed, MS peaks with a mass difference of as small as 3.4 mDa were well identified. A total of 12027, 15593, and 8029 peaks in the mass spectra of the water, aerosol, and soil samples, respectively, were assigned to known elemental formulae. Peaks Ⅱ and Ⅲ were hydrophilic components mainly including CHNO and CHO compounds. Compared with peak Ⅱ, peak Ⅲ exhibited a significant increase in CHNOS and CHOS, indicating that UPLC exerted a certain separation effect on these compounds. Furthermore, the aerosol samples contained a higher concentration of sulfur-containing compounds than the water and soil samples, primarily because of the abundance of organic sulfates present in atmospheric and cloud water.

Data processing and graphic visualization revealed that the unique components in the water samples mainly appeared in the area of 0.1<O/C<0.5 and 1.0 <H/C<1.7. The compounds detected were low-oxygen and highly condensed lignin-like compounds. The unique components in the aerosol samples appeared in the area of 0.4<O/C<1.0 and 1.5<H/C<2.0, and were classified as carbohydrates. The unique components in the hydrophilic fraction of the soil samples were found in the area of 0.6<O/C<1.0 and 0.5<H/C<1.0, and were determined to be tannin-like compounds. By contrast, the components in the hydrophobic fraction were similar to those found in the water samples and appeared in the region containing lignin-like compounds. In summary, this study proposed a novel analytical protocol to characterize DOM from different ecosystems using UPLC-FT-ICR MS. This method could separate DOM components using UPLC with eluents of different polarities and analyze them using high-resolution FT-ICR MS to reveal their molecular compositions and possible chemical types. This protocol offers solid technical support for the comprehensive profiling of DOM at the molecular level.

溶解性有机质(DOM)是由众多有机化合物组成的高度异质性混合物,普遍存在于湖泊、河流、海洋、大气气溶胶及土壤等地球表层环境中。DOM作为生物的营养物质,参与生物地球化学循环系统^[1]^,在塑造生态系统方面发挥着关键作用,并在调节水质、影响气候变化、污染物运输迁移等方面深刻影响着人类的生存环境^[2,3]^。因此,在分子水平上准确表征DOM的组成,对有效解析其在不同环境体系中的理化特征,进而阐明DOM的地球化学行为具有重要意义。

傅里叶变换离子回旋共振质谱(fourier transform ion cyclotron resonance mass spectrometry, FT-ICR MS)是目前从分子水平分析DOM组成最为有效的技术手段^[4]^。FT-ICR MS拥有超高分辨率和超高质量精确度,因此能够准确解析谱图中各质谱峰所对应的分子式^[5]^。FT-ICR MS虽然不能直接对分子进行结构解析,但可以通过分子式的元素比率和芳香度信息有效地分析DOM的组分特征,以便追踪DOM的来源、转化和归趋,研究DOM与微生物、自然介质之间的交互关系^[6]^。FT-ICR MS通常采取直接进样(direct injection, DI)的方式分析DOM,采用较长的采集时间和上百次的谱图叠加,以获得较高的质量分辨率,并提高谱图信噪比^[7]^。尽管DI模式易于操作且灵敏度高,但其易受电离歧视、基质效应以及金属离子加合物的影响,很难检测到低浓度、低极性和低电离效率的化合物^[8]^。另外,FT-ICR MS分析DOM另一个不足之处是只能得出元素的组成信息,无法区分同分异构化合物。上述问题可以通过在质谱分析之前先进行色谱分离来解决,即通过色谱将DOM中的化合物按照分子尺寸或极性大小依次洗脱后进入质谱,从而大大削弱电离抑制,并使得同分异构化合物得到一定程度的分离。

目前,高效液相色谱(HPLC)结合有机质谱用于DOM的研究,一般是通过耦合轨道阱(Orbitrap)质谱^[9]^或离子阱、四极杆等质谱仪器^[10,11]^。这类质谱在一个道尔顿的质量窗口内可鉴定出的质谱峰数目相较于FT-ICR MS明显偏少,而FT-ICR MS受限于高分辨率数据所需的采集时长,通常为2~8 s,无法与液相色谱(LC)的洗脱窗口相匹配,因此鲜有FT-ICR MS与LC在线研究的报道。随着ICR技术创新和商业推广,最新一代的FT-ICR MS配置四极检测(quadrupolar detection, QPD)技术^[12]^可在获取相同分辨率数据的条件下缩短一半的采集时间,使在线超高效液相色谱(UPLC)与FT-ICR MS联用成为可能。Kim等^[13]^首次通过分析腐殖酸标准品建立了在线LC FT-ICR MS分析方法,并从中成功分离出木质素和单宁酸组分。随后,Han等^[14]^使用LC FT-ICR MS和柱后反梯度技术,分析出富里酸标准品中的高极性组分,而该类组分在DI模式下未被检出。但因在线LC FT-ICR MS技术未得到足够商用支持,其在环境样品分析中未得到广泛应用。

本文采用UPLC与FT-ICR MS联用技术对水体、气溶胶和表层土壤样品中的DOM进行了分离和表征。UPLC将样品中的DOM按照极性强弱分离为不同的极性组分,再通过对不同组分的质谱数据进行统计和可视化解析,可更好地揭示环境样品的DOM分子组成。这有助于在多维度范围内评估环境样品中DOM分子的多样性,为进一步研究其地球化学过程提供更为精细的解析途径。

## 1 实验部分

### 1.1 仪器与试剂

Dionex Ultimate 3000超高效液相色谱仪(美国ThermoFisher公司), SolariX 2XR 7T傅里叶变换离子回旋共振质谱仪(德国Bruker Daltonik公司)。Milli-Q Advantage A10超纯水系统(德国Merck公司)。

乙腈为色谱纯(德国Merck公司);甲酸为质谱纯(德国Sigma公司)。萨旺尼河富里酸标样(suwannee river fulvic acid, SRFA,富里酸含量占90%~95%,美国腐殖酸协会), Bond Elut PPL固相萃取柱(500 mg, 6 mL, 美国Agilent公司)。

### 1.2 仪器条件

#### 1.2.1 UPLC条件

色谱柱为Acquity UPLC BEH C18柱(100 mm×1.0 mm, 1.7 μm, 13 nm, Waters),预柱为Acquity HSS T3 VanGuard柱(5 mm×2.1 mm, 1.8 μm, 10 nm, Waters)。流动相为含0.1%(v/v)甲酸的水(A)和含0.1%(v/v)甲酸的乙腈(B)。梯度洗脱程序:0~5 min, 0B; 5~11 min, 0B~95%B; 11~25 min, 95%B; 25~28 min, 95%B~0B; 28~30 min, 0B。流速为0.1 mL/min,进样量10 μL。将紫外波长设置为274 nm(UV_274_),在连续样品之间注入不含分析物的空白样品以监测基线。

#### 1.2.2 FT-ICR MS条件

采用电喷雾电离源负离子电离模式(ESI(-))电离,毛细管电压5.0 kV,端板偏置电压-500 V,雾化气体压力200 kPa,干燥气温度250 ℃,流速5.0 L/min。宽带模式(*m/z* 150~1000)下采集质谱数据,四极检测模式,瞬态域信号为2M字长,自由感应衰减(free induction decay, FID)信号长度为0.74 s,离子累计时间为0.030 s,单次扫描的总离子流色谱图(total ion chromatogram, TIC)强度在5.0×10^8^和1.0×10^9^之间。同时使用在线质量数校正模式锁定285.0993、313.0946、369.1211、425.1112、469.1381、497.1330、523.1489、523.1489、569.1552共9组质量数,用于实时校正LC-MS分析过程中的质量数偏移。

### 1.3 样品采集与前处理

水体样品(WS)采集自天津大学卫津路校区青年湖(117.17°E, 39.11°N),大气气溶胶样品(AS)采集自天津大学第16教学楼顶层,土壤样品(SS)采集自天津大学第16教学楼门口花坛,以上样品均在2021年12月份采集。气溶胶和土壤样品经纯水振荡萃取后,用0.45 μm孔径的玻璃纤维膜过滤,然后将其引入经甲醇和酸化超纯水(pH=2)预处理过的固相萃取柱内。待滤液全部经过萃取柱后,氮气吹干柱内水分,再用甲醇洗脱柱内有机质完成DOM富集实验^[15]^。最终样品以200 mg/L的质量浓度溶解在水-乙腈(1∶1, v/v)中进行UPLC-MS实验。

### 1.4 数据处理

UPLC-MS完成数据采集后,使用Compass DataAnalysis 5.0软件对原始数据集进行内部重新校准,选取*S/N*≥4、质量偏差小于0.6×10^-6^ (0.6 ppm)的质谱峰,使用本实验室开发的软件和自动选峰算法,完成目标化学式的赋值分配。其中,将C、H、N、O、S元素数目限定为^12^C(1~50)、^1^H(1~120)、^16^O(0~30)、^14^N(0~2)、^32^S(0~2);元素比限定为H/C<2.0和O/C<1.2。

## 2 结果与讨论

### 2.1 液相色谱分析

为了评估液相色谱分离性能,使用SRFA标样作为分离样品进行液相色谱分离,所得色谱图如图1所示。SRFA标样经色谱柱分离后,得到3个不同的色谱峰。第一个色谱峰的丰度相对较低,出峰时间在1.29~1.85 min处,是SRFA标样中极性最强的组分;丰度最高的色谱峰出现在1.85~5.12 min,是SRFA标样中的主体成分富里酸;最后一个色谱峰在13.95~28.05 min处洗脱,峰形较宽,呈明显的“驼峰”,为SRFA标样中的木质素组分^[13]^。

**图1:**
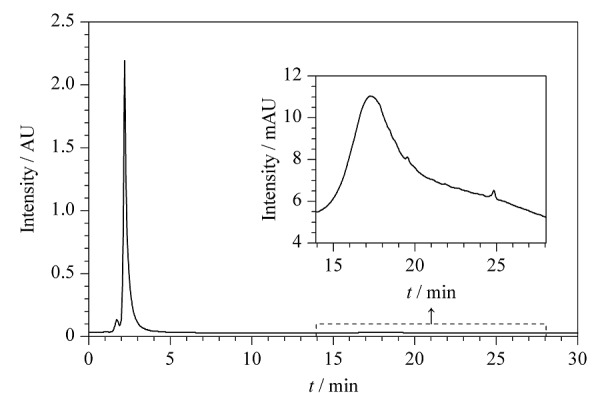
SRFA标样的液相色谱-紫外光谱图

受色谱柱尺寸和流速的限制,流动相携带样品在1.29 min到达紫外检测器。在1.29~5.00 min内,样品中极性最强的化合物被完全洗脱。5~11 min流动相由100%的水相匀速变为含5%水的有机相,此时UV_274_吸光度保持不变。直至洗脱液中乙腈含量达到95%后,最后一个洗脱峰才出现,但UV_274_吸光度极小,仅占洗脱主峰(1.85~5.12 min)强度的0.52%。随后UV_274_吸光度平稳下降,在29.08 min后洗脱峰强度下降至与初始位置相当,证实SRFA标样中所有溶解性组分均已完成洗脱。

### 2.2 UPLC-MS表征环境样品中溶解性有机质

#### 2.2.1 液相色谱分离表征

使用与检测SRFA标样相同的色谱方法,结合FT-ICR MS分别对水体、气溶胶和土壤样品进行了UPLC-MS分析。环境样品中DOM分子组成的复杂性高于SRFA标样,如图2所示。在0~6 min内,环境样品和SRFA标样内的亲水性组分被洗脱, 环境样品除了与SRFA标样有相似的色谱峰Ⅰ、Ⅱ(色谱保留时间分别为1.70~2.20 min和2.24~2.78 min)外,在色谱峰Ⅱ后还有明显的一个新色谱峰,记作色谱峰Ⅲ。环境样品类型不同,色谱峰Ⅲ的保留时间也略有不同。本次实验中水体、气溶胶、土壤样品色谱峰Ⅲ的保留时间分别为3.10~5.93 min、3.25~5.75 min和3.72~4.42 min。色谱峰Ⅲ是DOM中一个重要的极性组分。为更好地分离色谱峰Ⅱ、Ⅲ,在液相色谱柱之前安装有能够保留极性化合物的硅基键合相预柱(T3柱),以更好地提高极性组分的分离效果。在6~16 min之间,没有检测到化合物的洗脱。随后在16~21 min之间,95%有机相平衡洗脱5 min后,色谱图中出现了较宽的“驼峰”,记作色谱峰Ⅳ。色谱峰Ⅳ为样品体系中的疏水性组分,在气溶胶样品中的相对丰度最高,水样略低,土壤次之;而SRFA标样中色谱峰Ⅳ的丰度略高于本底基线,与图1数据吻合。

**图2:**
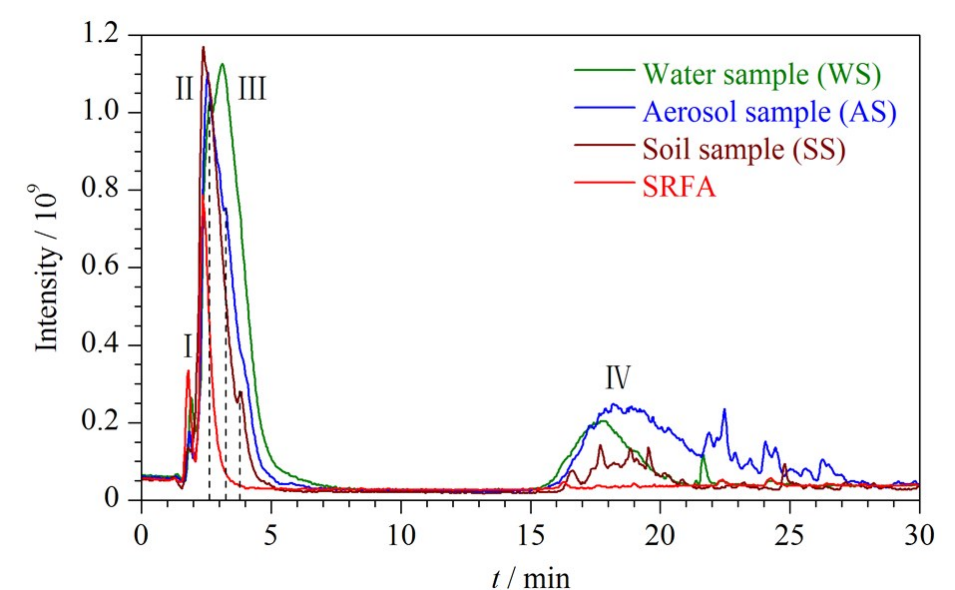
SRFA标样与3种环境样品的总离子流色谱图

#### 2.2.2 质谱峰分子式匹配

为获得环境样品更为全面的分子信息,先对环境样品进行UPLC分析,然后选取各自的Ⅰ、Ⅱ、Ⅲ、Ⅳ段色谱峰进行质谱分析,并对每个色谱峰生成相应的平均质谱图。图3为不同类型的环境样品中色谱峰Ⅰ的平均质谱图。在图中可以观察到一系列重复单位为Δ*m*=67.9872的质谱峰,已在图中做了质量数标记。通过解析这类化合物的精确质量数,并结合同位素精细结构分析,可以确定化合物离子为甲酸钠簇[(NaCOOH)*_n_*HCOO]^-^^[16]^。C18色谱柱是一种典型的非极性色谱柱,极性化合物相较于非极性化合物更易被洗脱,甲酸钠簇等结合金属离子的有机络合物极性较强,无法被UPLC柱保留,会在流动相到达的第一时间被洗脱出来。在电喷雾电离过程中,这些离子形成甲酸钠的倍聚离子峰。含盐组分的优先洗脱消除了强极性化合物对电离带来的干扰,有助于提升难电离化合物的电离效率,优化FT-ICR MS的检测效果,为各类环境样品中Ⅱ~Ⅳ组分的高效电离及相对分子质量的准确测定提供了可靠的保障。

**图3:**
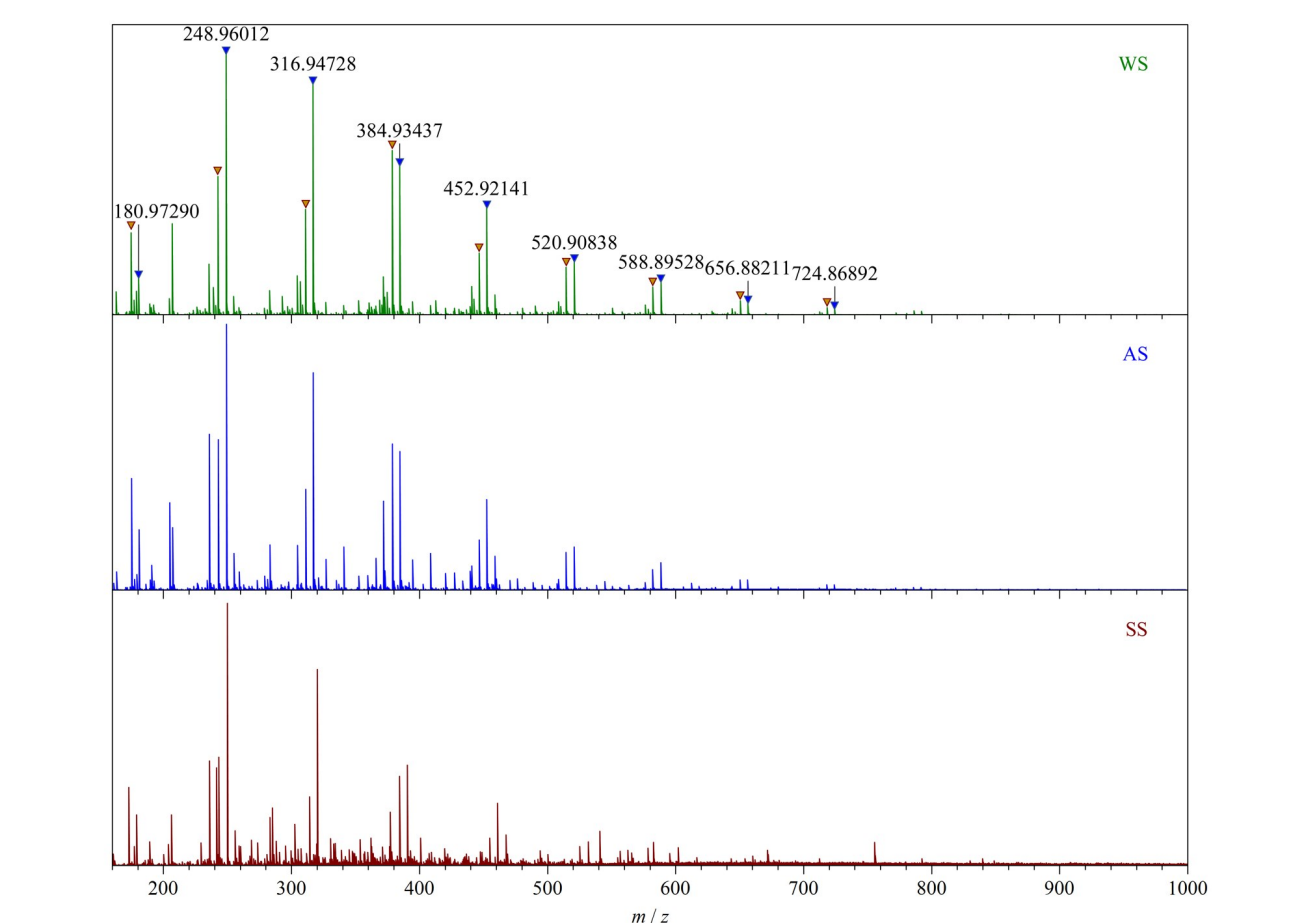
3种环境样品色谱峰Ⅰ的平均质谱图

图4为不同环境样品中色谱峰Ⅱ~Ⅳ的平均质谱图(左)。可以看出,水体样品中DOM的各段相对分子质量分布在*m/z* 200~800内,在*m/z* 400附近信号强度最高,呈正态分布;气溶胶样品中DOM相对分子质量分布范围较窄,在*m/z* 150~600内;而土壤样品中DOM分布最宽,在*m/z* 150~900均有分布,这与不同环境体系对DOM的富集能力密切相关。

**图4:**
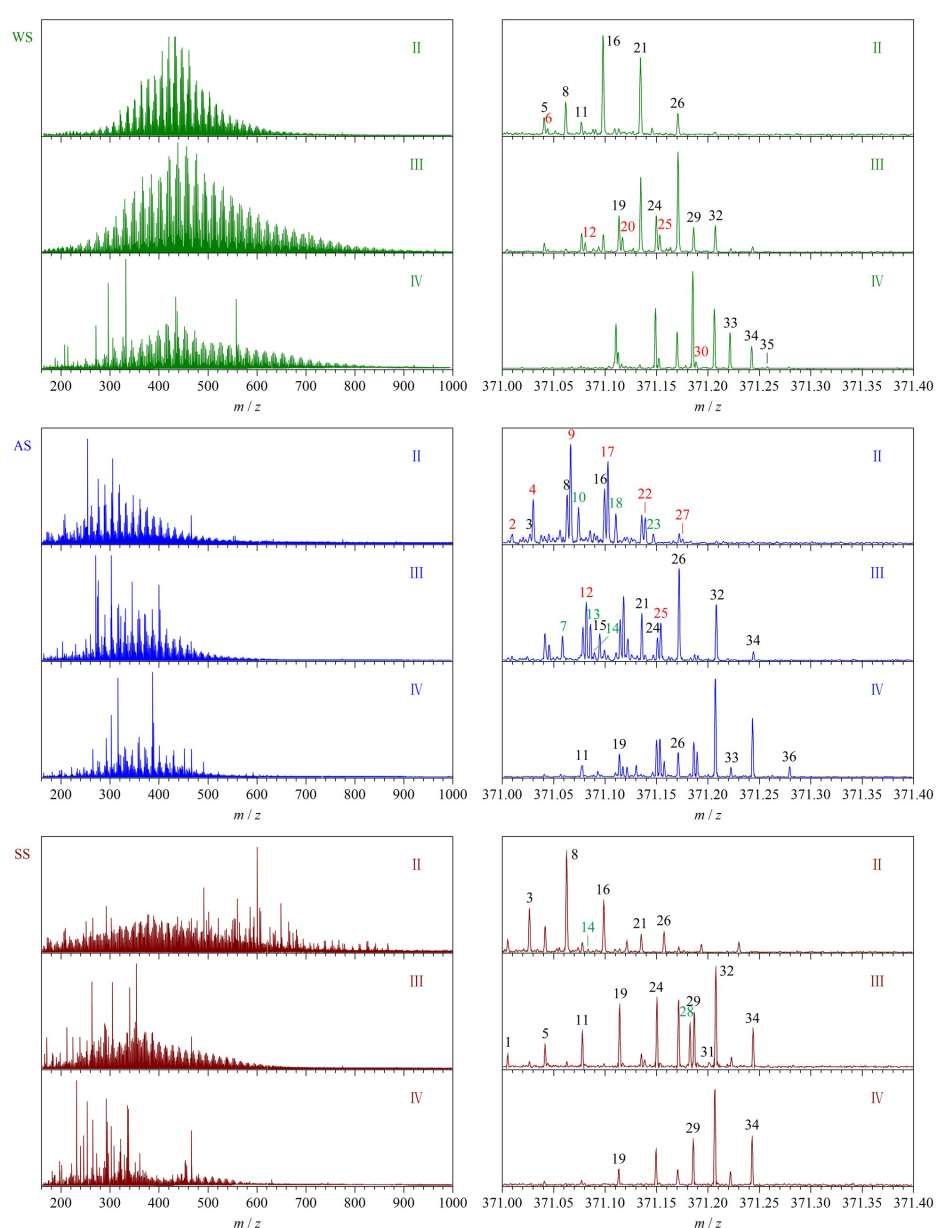
环境样品色谱峰Ⅱ~Ⅳ的平均质谱图(左)及*m/z* 371.00~371.40区域的放大图(右)

超高的质量分辨率是FT-ICR MS分析复杂环境样品的关键能力。在实际样品分析过程中,通过色谱分离,样品DOM组分依次洗脱,大幅降低了自身的复杂性,降低了不同极性化合物之间的重叠程度,提高了仪器检测的灵敏度。图4(右)为左图*m/z* 371.00~371.40区域内的放大图,根据C*_c_*H*_h_*N*_n_*O*_o_*S*_s_*(下标为原子个数)的形式对图中质谱峰进行化学式匹配,其中仅含CHO的化合物使用黑色序号,含CHOS和CHNO的化合物分别用红色序号和蓝色序号标记,详细的质谱峰定性数据见表1。黑红色数字连续且相差3.4 mDa的质谱峰可以完全分离,如峰5和6、峰16和17等^[17]^。

**表1:** 图4中*m/z* 371.00~371.40区域内的质谱峰定性表

No.	Compound	Meas. *m/z*	Theo. *m/z*	Error^*^/ 10^-6^	Resolution
1	C_17_H_7_O_10_	371.00449	371.00447	0.05	228435
2	C_14_H_11_O_10_S	371.00783	371.00784	-0.03	219409
3	C_14_H_12_O_12_	371.02562	371.02560	0.05	238041
4	C_11_H_16_O_12_S	371.02902	371.02897	0.13	219930
5	C_18_H_12_O_9_	371.04085	371.04086	-0.03	230186
6	C_15_H_16_O_9_S	371.04425	371.04423	0.05	222517
7	C_10_H_16_N_2_O_13_	371.05789	371.05796	-0.19	235696
8	C_15_H_16_O_11_	371.06194	371.06198	-0.12	222111
9	C_12_H_20_O_11_S	371.06537	371.06536	0.03	233484
10	C_14_H_16_N_2_O_10_	371.07321	371.07322	-0.03	221629
11	C_19_H_16_O_8_	371.07718	371.07724	-0.16	238796
12	C_16_H_20_O_8_S	371.08062	371.08061	0.03	218654
13	C_22_H_16_N_2_O_2_S	371.08597	371.08597	0.00	247183
14	C_18_H_16_N_2_O_7_ O_8_S_2_	371.08843	371.08847	-0.11	235401
15	C_23_H_16_O_5_	371.09446	371.09453	-0.19	236362
16	C_16_H_20_O_10_	371.09834	371.09837	-0.08	232788
17	C_13_H_24_O_10_S	371.10176	371.10174	0.05	218456
18	C_15_H_20_N_2_O_9_	371.10963	371.10960	0.08	271715
19	C_20_H_20_O_7_	371.11359	371.11363	-0.11	258754
20	C_17_H_24_O_7_S	371.11698	371.11700	-0.05	227070
21	C_17_H_24_O_9_	371.13477	371.13476	0.03	243303
22	C_14_H_28_O_9_S	371.13810	371.13813	-0.08	233750
23	C_16_H_24_N_2_O_8_	371.14595	371.14599	-0.11	227209
24	C_21_H_24_O_6_	371.14999	371.15001	-0.05	220312
25	C_18_H_24_O_6_S	371.15337	371.15338	-0.03	244303
26	C_18_H_28_O_8_	371.17114	371.17114	0.00	242029
27	C_15_H_32_O_8_S	371.17450	371.17451	-0.03	239203
28	C_17_H_28_N_2_O_7_	371.18232	371.18237	-0.13	249434
29	C_22_H_28_O_5_	371.18640	371.18640	0.00	237011
30	C_19_H_32_O_5_S_1_	371.18771	371.18769	0.05	227815
31	C_26_H_28_O_2_	371.20167	371.20165	0.05	241226
32	C_19_H_32_O_7_	371.20751	371.20753	-0.05	246937
33	C_23_H_32_O_4_	371.22277	371.22278	-0.03	242902
34	C_20_H_36_O_6_	371.24389	371.24391	-0.05	223306
35	C_24_H_36_O_3_	371.25915	371.25917	-0.05	245639
36	C_21_H_40_O_5_	371.28033	371.28030	0.08	241609

* Error=(Meas. *m/z*-Theo. *m/z*)/Theo. *m/z*.

### 2.3 可视化数据表征

FT-ICR MS特别适合解析环境样品中复杂的有机质,其独有的超高分辨率和质量精度可以在分子水平上详细表征混合物。本次实验在水体、气溶胶和土壤样品中检测到上万个独立的质谱峰,并对其进行化学式匹配。经统计水体样品中有效匹配分子式的质谱峰数目为12027个,气溶胶样品和土壤样品分别为15593个和8029个,相关统计不含同位素峰对应的化学式。具体化学式数目详见图5(左)。如此繁多的数据信息需要采用更为直接的可视化表示方法对数据整合归纳,以便更好地理解数据之间的相互关系,总结分子特征,归纳变化规律。

**图5:**
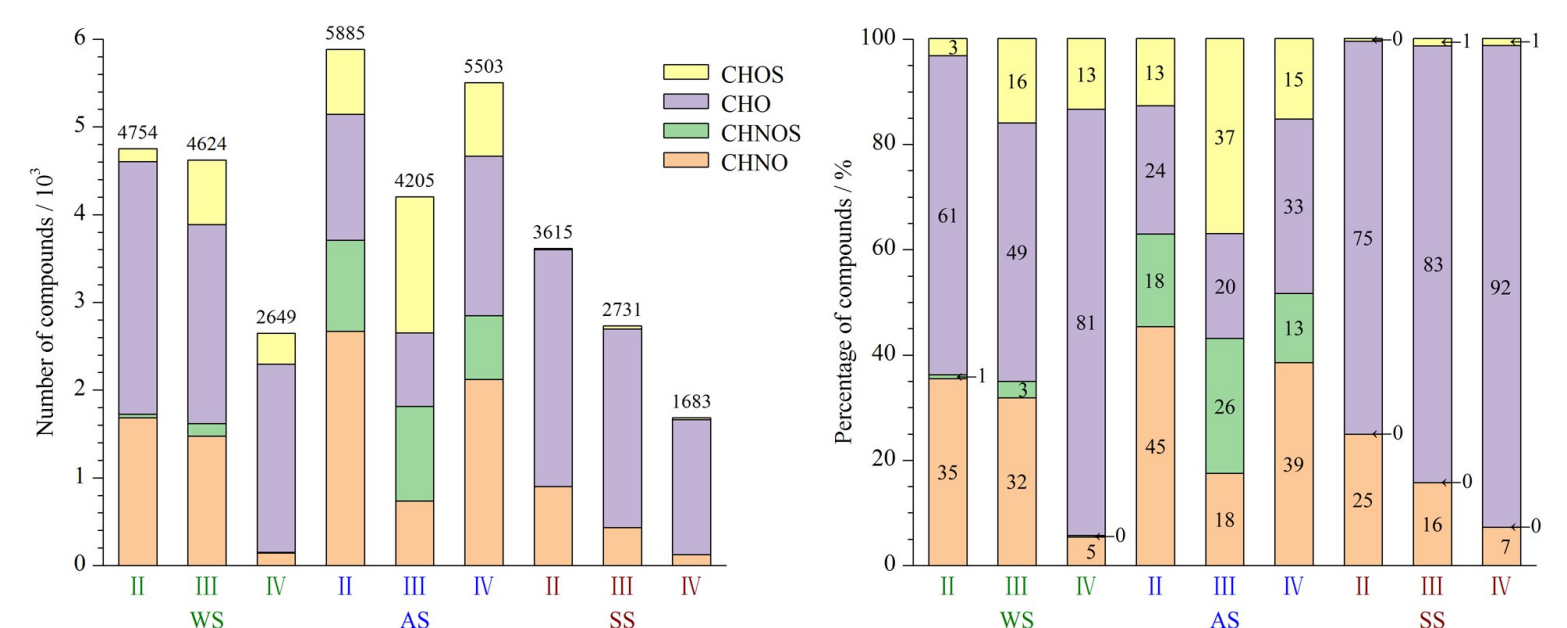
3种环境样品中4类化合物相关分子式数量及归一化分布图

图5为色谱峰Ⅱ~Ⅳ中化合物类型分布图,左图纵轴表示化合物数,右图纵轴为化合物数百分比,是将所鉴定出的化合物数目做归一化处理,是左图的补充。由图5可知,在水体和土壤样品中,分子式匹配结果为CHO类化合物的数目最多,其丰富的羟基和羧基基团是DOM的重要分子特征,且基团在ESI(-)模式下有较好的电离效果。ESI(-)会优先电离DOM中电负性强的酸性化合物。

Ⅱ、Ⅲ馏分段均是含0.1%甲酸的水洗脱出的亲水性组分,主要为CHNO和CHO类化合物,与馏分Ⅱ相比,馏分Ⅲ中CHNOS和CHOS类化合物数目有显著提高,说明UPLC对其有一定的分离作用。水体和土壤样品分布相似,在UPLC洗脱过程中,随着流动相的变化,CHNO类化合物的数目逐渐减少。这是由于大多的含氮化合物是亲水性化合物,更容易被水相洗脱。气溶胶样品化合物数目最多,且拥有明显多于水体样品和土壤样品的含硫化合物,这主要是与大气和云雾水中大量存在的有机硫酸盐有关^[18]^。

值得注意的是,经过UPLC分离,相同相对分子质量不同结构式的化合物会出现在不同的色谱组分中,这有助于FT-ICR MS鉴定出更多的同分异构体化合物。

图6为环境水体样品色谱峰Ⅱ~Ⅳ中O*_x_*和O*_x_*S类化合物的柱状分布图,纵坐标为相应质谱峰的丰度值。其中,O*_x_*和O*_x_*S类化合物分别是指含有*x*个氧元素的CHO类和CHOS类化合物。O*_x_*类化合物主要分布在色谱峰Ⅱ中,但主要类型分布略有不同。土壤样品的含氧化合物主要在O_1_~O_20_范围内呈正态分布,O_11_处为最高值,分布范围最广;水体样品在O_1_~O_17_分布,O_10_最高;气溶胶样品在O_1_~O_15_分布,O_9_最高,分布范围最小。在色谱峰Ⅲ中亦存在含量较为丰富的O*_x_*类化合物,但其类型和分布均少于色谱峰Ⅱ。在色谱峰Ⅲ内,O*_x_*S类化合物峰值最高,有显著的富集趋势,在O_3_S~O_7_S处尤为明显。色谱峰Ⅳ与Ⅱ和Ⅲ相比存在明显的差异,Ⅳ中O*_x_*S化合物种类明显少于前两者而且含氧原子的数目偏低。说明在富氧的含硫化合物中富含更多的羧基基团,有着更强的亲水性,更容易被水洗脱。

**图6:**
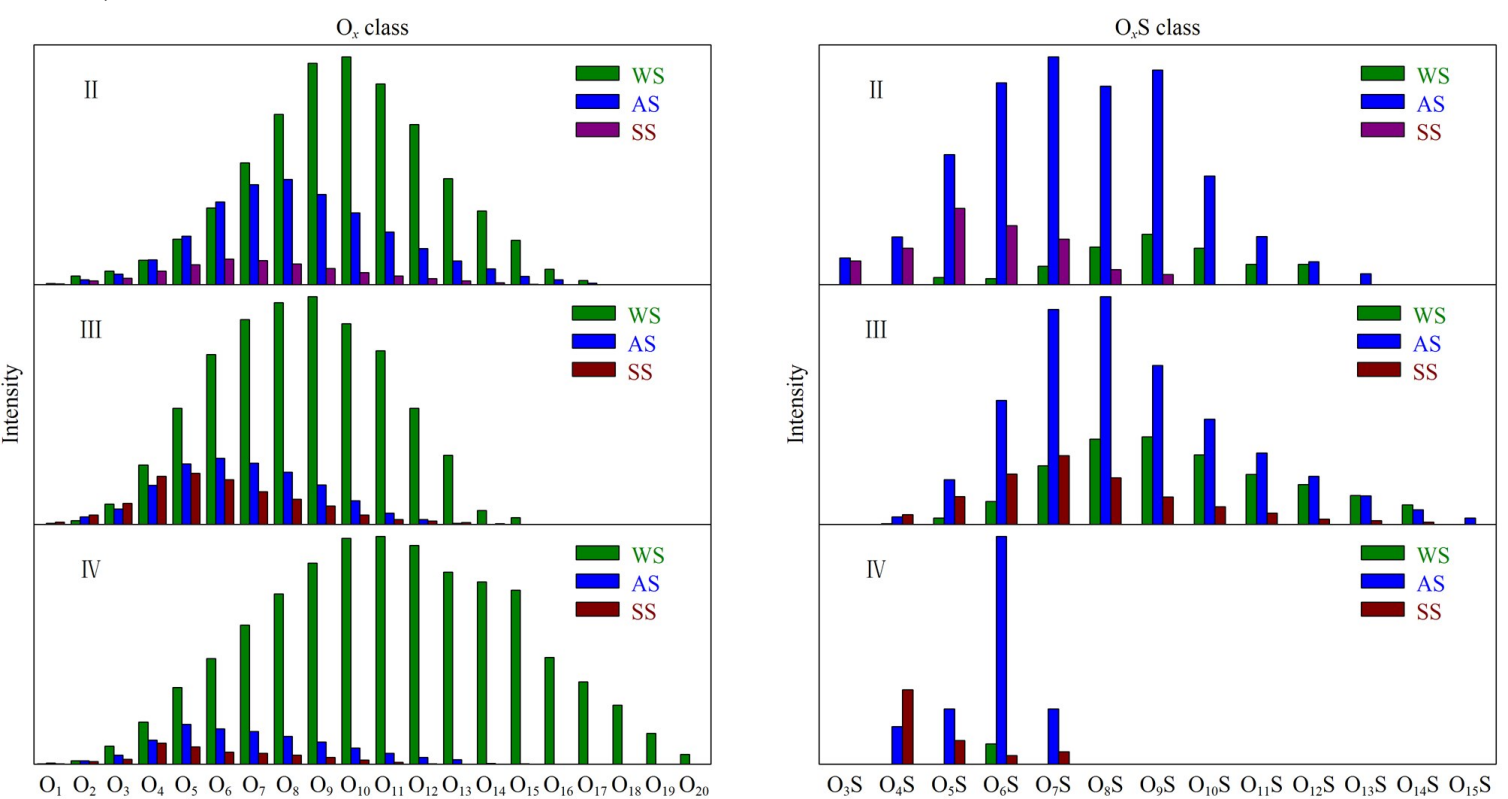
3种环境样品中O*_x_*和O*_x_*S类化合物的相对丰度分布图

为了进一步分析每个UPLC色谱峰的分子组成,将环境样品色谱峰Ⅱ~Ⅳ检测出的化合物进行的范克莱夫伦(van Krevelen, vK)图绘制,如图7。根据DOM中的H/C、O/C和改进芳香度指数(aromatic index, AI_mod_), vK图分为7个区域并对应DOM中脂类、肽类/蛋白类、脂肪族类、碳数化合物类、不饱和烃类、木质素类、稠环芳烃类和单宁酸类7种化合物类型^[19]^。AI_mod_可参照公式AI_mod_=(1+*c-*0*.*5*o-s-*0*.*5*h*)*/*(*c-*0*.*5*o-s-n*)计算,式中*c*、*h*、*o*、*n*和*s*是指化学式中C、H、O、N、S元素的数目。图中点的面积越大,颜色越深表示该化合物的相对丰度值越高。其中,单宁酸类(tannin-like)化合物分布在0.6<O/C<1.2、0.5<H/C<1.5、AI_mod_<0.67区域;木质素类(lignin-like)分布在0.1<O/C<0.6、0.6<H/C<1.7、AI_mod_<0.67区域;蛋白质/氨基糖类(protein-like)分布在0.2<O/C<0.6、1.5<H/C<2.2区域;多糖(carbohydrates)分布在0.6<O/C<1.2、1.5<H/C<2.2区域。在色谱峰Ⅱ、Ⅲ中,单宁酸类化合物占主导地位;随着洗脱液的变化,水相逐渐减少,极性降低,导致色谱峰Ⅳ的vK图的中心偏移到左上角,H/C比增加、O/C比降低,高饱和度的木质素类在水体和土壤样品中得到洗脱,而气溶胶样品中以蛋白质/氨基糖类化合物为主。

**图7:**
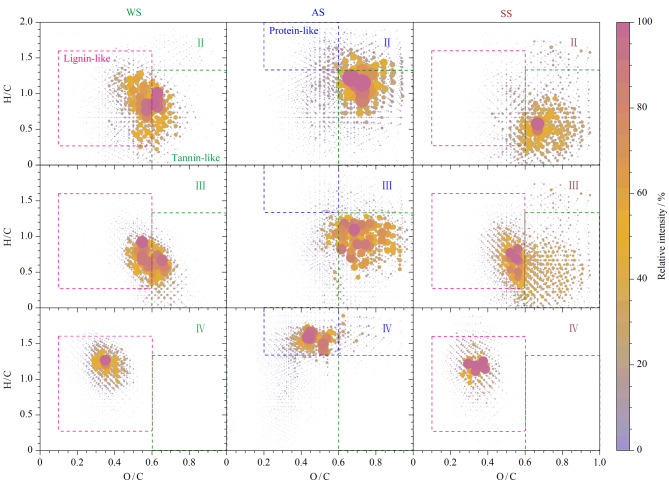
3种环境样品色谱峰Ⅱ~Ⅳ范克莱夫伦图

图8为环境样品的vK差异图,作为图7的补充,主要用于区分不同环境样品中相同位置色谱峰的组成差异性。其中,浅蓝色为3种环境样品的共有化合物,在色谱峰Ⅱ、Ⅲ、Ⅳ中的数目分别为2876、2546、932;绿色、黄色和棕色分别表示水体、气溶胶和土壤样品中的特有组分。在UPLC洗脱过程中,水体样品的特有组分主要出现在0.1<O/C<0.5、1.0<H/C<1.7区域,为低氧高饱和度的木质素组分;气溶胶样品的特有组分出现在0.4<O/C<1.0、1.5<H/C<2.0区域,属于糖类化合物的分布范围;与之不同的是,土壤样品在极性洗脱段(色谱峰Ⅱ和Ⅲ)的特有组分出现在0.6<O/C<1.0、0.5<H/C<1.0区域,是单宁酸类化合物,而在非极性洗脱段的特有组分与水体样品相似,出现在木质素区域。上述分析表明,水体中有较多的木质素类化合物,气溶胶存在更为丰富的碳水化合物,而土壤中特有的单宁酸类化合物较多。与水体和土壤样品相比,气溶胶DOM在vK图中的化合物类型及化学多样性要高出很多。

**图8:**
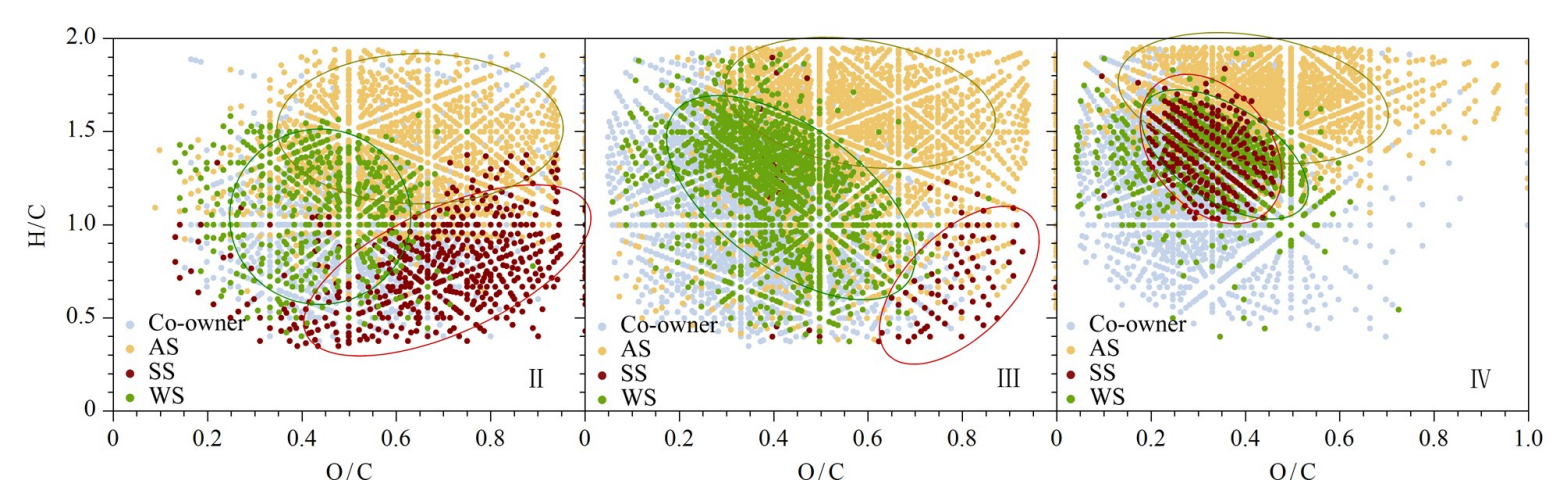
3种环境样品色谱峰Ⅱ~Ⅳ的范克莱夫伦差异图

图9为环境样品色谱峰Ⅱ、Ⅳ中亲水/疏水性化合物的分布图^[20]^,用于分析DOM分子的水溶性。以分子的平均碳氧化态(nominal oxidation state of carbon, NOSC)为纵坐标,等效双键(double-bond equivalence, DBE)为横坐标,绘制出DOM的高级结构的轮廓。NOSC和DBE分别参照公式NOSC=4-[(4*c+h-*3*n-*2*o-*2*s-z*)*/c*]和DBE=(2*c+*2*-h+n*)/2计算,式中*z*为所带电荷。

**图9:**
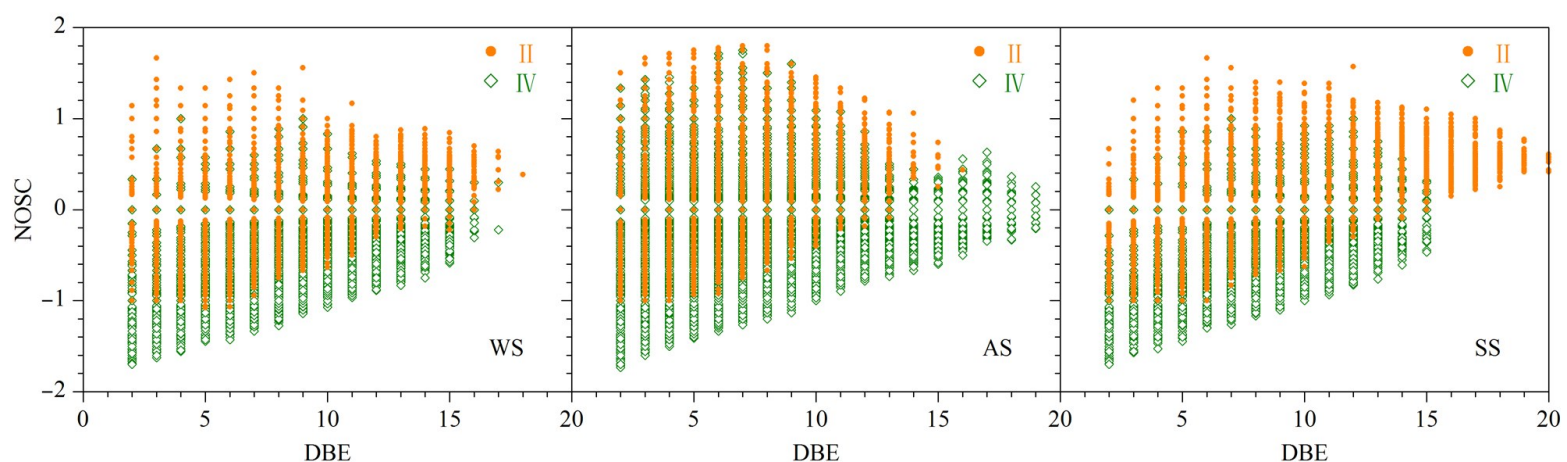
3种环境样品色谱峰Ⅱ和Ⅳ的亲水/疏水性化合物分布图

在所有环境样品的色谱峰Ⅱ中NOSC为正值的占主导地位,这表明其极性较强,亲水性较高,更容易溶于水;而色谱峰Ⅳ中NOSC为负值的占比最多,结果与色谱柱的选择性一致,其化合物的极性越低,整体疏水性越高,越不易被水洗脱。不同环境样品的缩合度相差明显,水体样品在色谱峰Ⅱ、Ⅳ中分子缩合度基本相同,而气溶胶样品疏水性组分有更高的缩合度,土壤样品与之相反,高缩合度的是亲水性组分。这些结果表明,UPLC使样品DOM中亲水性分子和疏水性分子得到有效分离;土壤DOM中高缩合度的亲水性化合物易于向地下水体系中释放;而气溶胶DOM与之相反,高缩合度的疏水性化合物使其不易被雨水或云雾气溶解。

## 3 结论

本研究建立了一种通过在线UPLC和FT-ICR MS联用表征环境样品DOM复杂性和多样性的方法。UPLC为质谱分析增加了一个极性维度信息,而没有增加太多的分析时间,可以很好地从复杂的DOM中分离出盐、单宁酸、木质素和蛋白/氨基糖等特征组分,使在常规测试条件下难以被ESI电离的木质素和蛋白/氨基糖类化合物得到更加完整的表征。从色谱峰的保留时间及vK图中共有化合物的数目可知,DOM在不同环境中具有相似的色谱行为和化合物类型,同时也展现出丰富的化学多样性信息。不同的是,气溶胶样品中含有更为丰富的含硫、含氮化合物,而水体和土壤样品中木质素和单宁酸类化合物更多。为更深入地表征DOM的分子结构信息,后续工作将尝试在UPLC-MS的基础上引入离子碎裂技术,为复杂DOM样品中标志性污染物的识别、跟踪提供可行的技术手段。
